# A mitochondrial regulatory network of ferroptosis defense in HPV-positive cervical cancer: therapeutic implications of the mitoSTAT3-DHODH axis

**DOI:** 10.3389/fphar.2026.1868059

**Published:** 2026-06-17

**Authors:** Shi Feng, Yang Huang, Zhi Li, Zhanxu He, Yuhong Gao

**Affiliations:** Central Hospital Affiliated to Shenyang Medical College, Shenyang, Liaoning, China

**Keywords:** ferroptosis, HPV-positive cervical cancer, lipid peroxidation, mitoSTAT3-DHODH axis, oxidative stress

## Abstract

Human papillomavirus (HPV)-positive cervical cancer develops under persistent metabolic, replicative, and oxidative stress and frequently exhibits limited sensitivity or acquired resistance to cisplatin-based concurrent chemoradiotherapy (CCRT). In this context, ferroptosis represents a potential therapeutic vulnerability, yet the regulatory networks that sustain ferroptosis resistance in HPV-positive tumors remain incompletely defined. This review proposes a mitochondria-centered framework in which mitochondrial STAT3 (mitoSTAT3), electron transport chain (ETC)-dependent coenzyme Q (CoQ) redox turnover, and dihydroorotate dehydrogenase (DHODH) may form a context-dependent regulatory axis of ferroptosis defense. We first summarize how HPV E6 and E7 oncoproteins impose chronic biosynthetic, replicative, and oxidative pressures that increase tumor dependence on mitochondrial redox and bioenergetic control. We then discuss STAT3 not only as a canonical nuclear transcription factor but also as a stress-adaptive signaling node with mitochondria-associated functions. Current evidence suggests that mitoSTAT3 may support ETC efficiency, limit electron leakage, and help maintain CoQ cycling, whereas DHODH may contribute to localized CoQH_2_-dependent radical-trapping activity within the inner mitochondrial membrane. Together, these mechanisms may restrain mitochondrial lipid peroxidation and thereby reduce susceptibility to ferroptotic injury. Finally, we discuss the translational implications of this framework, including ferroptosis-sensitizing strategies, DHODH inhibition, STAT3-directed interventions, biomarker-guided patient stratification, rational combinations with chemoradiotherapy or immunotherapy, and nanomedicine-enabled delivery. Overall, this review identifies the mitoSTAT3-DHODH axis as a mechanistically plausible regulatory network of ferroptosis resistance and a potential therapeutic vulnerability in HPV-positive cervical cancer.

## 1 Introduction

In 2022, cervical cancer accounted for approximately 662,000 new cases and 349,000 deaths worldwide (Li Z. et al., 2025), underscoring its substantial burden among women. For patients with locally advanced or recurrent disease, cisplatin-based concurrent chemoradiotherapy (CCRT) remains the standard treatment (Chang and Miao, 2023). However, the true clinical challenge lies in the limitations of this standard therapy, as a significant proportion of these tumors exhibit primary resistance, rapid recurrence, and acquired tolerance (Zhang Hanqun et al., 2023). Cytotoxic therapy disrupts the cellular redox milieu, inducing reactive oxygen species (ROS), destabilizing membranes, and causing chronic oxidative stress (Bhardwaj and He, 2020). Rather than merely acting as a byproduct of treatment, oxidative stress exerts strong selective pressure, and surviving cells adapt by leveraging metabolic plasticity to upregulate their antioxidant defense capacity (Bhardwaj and He, 2020; Pendleton et al., 2023). By sustaining high nicotinamide adenine dinucleotide phosphate (NADPH) pools and anchoring the mitochondrial redox balance, these malignancies may evade ROS-induced cell death (Pendleton et al., 2023; Tang et al., 2022; Ju et al., 2020; Foo et al., 2021).

Therapy resistance should be recognized as a dynamic state orchestrated by metabolic flux and spatially compartmentalized redox defenses (Gonçalves et al., 2021). Many anticancer therapies rely on the induction of apoptosis; however, HPV-positive cervical cancer cells do not always respond through this route (Pal and Kundu, 2019). Human papillomavirus (HPV) oncoproteins E6 and E7 undermine key apoptotic safeguards through E6-mediated suppression of tumor protein p53 (p53) signaling and E7-driven deregulation of cell cycle control (Pal and Kundu, 2019; Kusakabe et al., 2023). With apoptotic responsiveness attenuated, ferroptosis, an iron-dependent form of regulated cell death driven by lipid peroxidation (LPO), emerges as a particularly relevant vulnerability (Chang and Miao, 2023; Wang et al., 2022).

The metabolic architecture of HPV-positive cervical cancer creates a paradox (Li and Sui, 2021; Pappa et al., 2021). HPV-driven malignancies exhibit hyperactive metabolic flux and high biosynthetic demand, a metabolic state that is expected to increase the oxidative burden and increase susceptibility to LPO-driven ferroptotic injury (Chang and Miao, 2023; Pappa et al., 2021). Despite this intrinsic vulnerability, HPV-positive cervical cancer cells often persist under chronic oxidative stress and withstand ROS-intensive chemoradiotherapy (Chang and Miao, 2023; Wang et al., 2022). This apparent resilience suggests that survival may depend on compartment-specific antioxidant defenses (Wang et al., 2023; Li et al., 2025b). Disruption of these protective systems lowers ferroptotic tolerance, thereby driving excessive LPO and ferroptotic collapse (Wang et al., 2022; Wang et al., 2023). Therefore, the goal is not to escalate ROS indiscriminately but to overwhelm ferroptosis tolerance in stressed cells by disabling specific mitochondrial defenses (Wang et al., 2023; Liu Y. et al., 2023). Signal transducer and activator of transcription 3 (STAT3) is widely known as a nuclear transcription factor that drives proliferation and immune evasion (Strobel et al., 2023). However, this canonical view does not fully explain how HPV-positive tumors endure chronic oxidative stress (Strobel et al., 2023; Chun et al., 2020).

In this review, we focus on a distinct non-canonical modality, mitochondrial STAT3 (mitoSTAT3). Driven by Ser727-centered stress signaling, a fraction of STAT3 translocates to mitochondria, where it regulates bioenergetics, electron leakage, and membrane potential stability (Conway et al., 2022; Piao et al., 2020; Lahiri et al., 2021). Dihydroorotate dehydrogenase (DHODH), an inner mitochondrial membrane (IMM) enzyme, couples metabolism to the coenzyme Q (CoQ) pool by reducing ubiquinone to ubiquinol (CoQH_2_), thereby contributing to local radical-trapping activity against LPO (Liu Y. et al., 2023; Lee and Roh, 2025). Current evidence suggests that mitoSTAT3-mediated bioenergetic regulation may be closely linked to this mitochondrial ferroptosis-defense mechanism (Liu Y. et al., 2023; Lahiri et al., 2021). Importantly, this mitochondrial protective capacity is not autonomous but depends on electron transport chain (ETC)-driven CoQ redox turnover (Lahiri et al., 2021).

**FIGURE 1:**
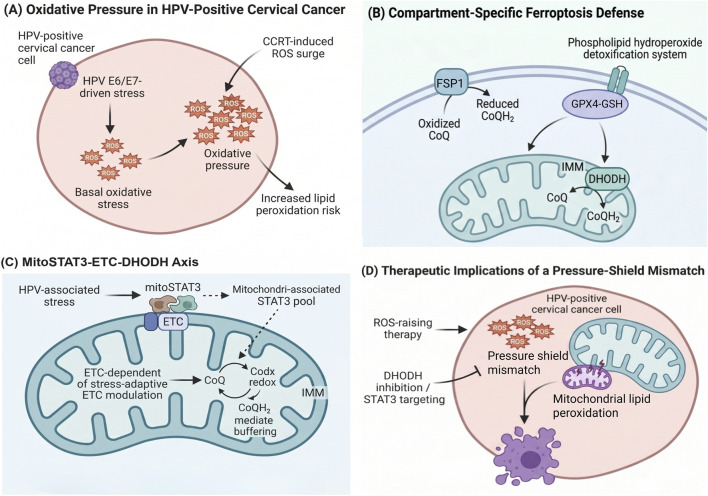
Overview of oxidative stress, compartment-specific ferroptosis defense, and the mitoSTAT3–ETC–DHODH framework in HPV-positive cervical cancer. **(A)** HPV-positive cervical cancer cells exist under chronic oxidative pressure, which is further intensified by cisplatin-based chemoradiotherapy and radiation, thereby increasing ROS stress and the risk of LPO. **(B)** Ferroptosis defense is spatially partitioned across membrane compartments. The GPX4–GSH system provides broad antioxidant protection, whereas FSP1 supports plasma membrane-localized CoQ reduction, and DHODH supports IMM-localized CoQH_2_ generation, thereby contributing to compartment-specific radical-trapping capacity. **(C)** Working model of the proposed mitoSTAT3–ETC–DHODH interface. In this framework, HPV-associated stress may favor a mitochondria-associated STAT3 fraction, referred to as mitoSTAT3, which may help support ETC efficiency and CoQ redox turnover. This, in turn, may help sustain DHODH-linked CoQH_2_-dependent radical-trapping protection and reduce susceptibility to mitochondrial ferroptotic injury. **(D)** Translational implications of the framework. Under ROS-inducing therapy, inhibition of DHODH may create a pressure–shield mismatch by weakening local mitochondrial defenses against LPO, thereby increasing susceptibility to ferroptotic cell death. Treatment-related immune collateral damage, particularly involving CD8^+^ T cells, may require monitoring and mitigation during therapeutic development. In this proposed model, HPV-associated stress promotes oxidative pressure; mitoSTAT3 may support ETC-linked CoQ redox cycling; DHODH-linked CoQH_2_ protection may restrain mitochondrial LPO; and disruption of this protection under ROS-raising therapy may promote ferroptotic cell death. Abbreviations: HPV, human papillomavirus; STAT3, signal transducer and activator of transcription 3; mitoSTAT3, mitochondrial STAT3; ETC, electron transport chain; DHODH, dihydroorotate dehydrogenase; CoQ, coenzyme Q (ubiquinone); CoQH_2_, ubiquinol; ROS, reactive oxygen species; LPO, lipid peroxidation; IMM, inner mitochondrial membrane; GPX4, glutathione peroxidase 4; GSH, glutathione; FSP1, ferroptosis suppressor protein 1; CCRT, cisplatin-based concurrent chemoradiotherapy. Created in BioRender. Huang, Y. (2026) https://BioRender.com/lpwqh8f.

Taken together, these observations support a mitochondria-centered framework in which mitoSTAT3, ETC-dependent CoQ cycling, and DHODH-mediated CoQH_2_-dependent protection function as an integrated defense axis in HPV-positive cervical cancer (Chang and Miao, 2023; Liu Y. et al., 2023). Disruption of this axis may weaken mitochondrial redox protection, increase susceptibility to therapy-induced LPO, and improve tumor response to ROS-generating treatments (Liu Y. et al., 2023; Lee and Roh, 2025). Therefore, defining this metabolic dependency may provide a mechanistic basis for new therapeutic strategies, including rational combination treatment and nanomedicine-enabled sensitization, in HPV-positive cervical cancer (Lee and Roh, 2025). The proposed conceptual framework is summarized in Figure 1.

## 2 Ferroptosis in cervical cancer: regulatory mechanisms, resistance, and mitochondrial relevance

Ferroptosis is a regulated form of iron-dependent cell death driven by LPO and failure of membrane-protective redox systems (Chang and Miao, 2023; Li et al., 2025b; Liu Y. et al., 2023). In cervical cancer, ferroptosis has gained attention because HPV-positive tumors often operate under persistent oxidative stress while also showing attenuated apoptotic responsiveness (Chang and Miao, 2023; Pal and Kundu, 2019; Kusakabe et al., 2023; Wang et al., 2022; Wang et al., 2023; Li et al., 2025b; Liu Y. et al., 2023). This combination creates a biologically meaningful paradox: the same oxidative pressure that should sensitize cells to LPO-mediated death may also select for tumor cells with stronger ferroptosis-defense capacity (Chang and Miao, 2023; Wang et al., 2022; Wang et al., 2023; Li et al., 2025b; Liu Y. et al., 2023).

Available evidence supports the role of canonical ferroptosis regulators in cervical cancer, including glutathione peroxidase 4 (GPX4), solute carrier family seven member 11 (SLC7A11), ferroptosis suppressor protein 1 (FSP1), mitochondrial ferroptosis-related proteins, and LPO markers (Chang and Miao, 2023; Wang et al., 2023; Li et al., 2025b; Liu Y. et al., 2023). These regulators should not be viewed as isolated modules. The GPX4-glutathione (GSH) system broadly reduces phospholipid hydroperoxides, SLC7A11 supports cystine import and GSH synthesis, FSP1 regenerates reduced CoQ at the plasma membrane, and DHODH contributes to mitochondrial CoQH_2_ generation at the inner mitochondrial membrane (Li et al., 2025b; Liu Y. et al., 2023; Lee and Roh, 2025). Their relative importance may differ according to membrane compartment, oxidative stress intensity, cysteine availability, mitochondrial load, and GPX4 status.

### 2.1 Ferroptosis and iron-redox vulnerability in cervical cancer

Ferroptosis sensitivity in cervical cancer is not merely a consequence of elevated ROS production but also reflects the convergence of iron availability, lipid-peroxidation pressure, and membrane-protective redox capacity (Chang and Miao, 2023; Wang et al., 2022; Li et al., 2025b; Liu Y. et al., 2023). HPV-positive cervical cancer develops in a biological context marked by persistent oxidative stress, altered metabolic flux, and impaired apoptotic responsiveness (Chang and Miao, 2023; Pal and Kundu, 2019; Kusakabe et al., 2023; Wang et al., 2022; Wang et al., 2023; Li et al., 2025b; Liu Y. et al., 2023). These features may make ferroptosis a biologically plausible alternative vulnerability in this setting. In addition, cervical cancer ferroptosis has been linked to iron-handling regulators, including transferrin receptor 1 (TFR1), ferritin heavy chain 1 (FTH1), and regulators of the labile iron pool (Chang and Miao, 2023; Wang et al., 2022; Li et al., 2025b). These observations indicate that iron availability should be considered together with ROS burden and LPO when evaluating ferroptosis vulnerability in cervical cancer.

This perspective is important because HPV-positive tumor cells may experience a dual pressure: increased metabolic and replicative demand can raise ROS and LPO burden, whereas cells that persist under this pressure may be enriched for stronger ferroptosis-defense mechanisms (Chang and Miao, 2023; Wang et al., 2022; Wang et al., 2023; Li et al., 2025b; Liu Y. et al., 2023). Therefore, ferroptosis resistance in cervical cancer should not be interpreted simply as the absence of oxidative stress. Rather, it may reflect the ability of tumor cells to prevent lipid-radical propagation within vulnerable membrane compartments under stress.

### 2.2 Why mitochondrial ferroptosis defense matters

The GPX4-GSH and SLC7A11 systems provide broad cytosolic and membrane protection, whereas FSP1 primarily supports plasma membrane-localized CoQ reduction (Li et al., 2025b; Liu Y. et al., 2023; Lee and Roh, 2025). By contrast, DHODH is positioned at the IMM and can couple pyrimidine metabolism to local CoQH_2_-dependent radical-trapping protection (Liu Y. et al., 2023; Lee and Roh, 2025). This topological distinction suggests that mitochondrial ferroptosis defense may remain functionally important even when canonical cytosolic antioxidant systems remain functional.

In HPV-positive cervical cancer, mitochondrial protection may be particularly relevant because viral oncoprotein-driven biosynthetic and replicative pressure can increase mitochondrial workload, electron flux, and the risk of local ROS leakage (Ortiz-Pedraza et al., 2023; Gore et al., 2025; Tsoneva and Yordanov, 2025). Under these conditions, the IMM functions as both a bioenergetic platform and a vulnerable membrane compartment exposed to redox stress (Liu Y. et al., 2023; Lee and Roh, 2025). Therefore, the DHODH-CoQ layer should not be viewed simply as an extension of cytosolic antioxidant defense. Rather, it may represent a spatially restricted mitochondrial defense module that helps limit LPO at sites of concentrated ETC-linked oxidative pressure (Li et al., 2025b; Liu Y. et al., 2023; Lee and Roh, 2025). This provides the rationale for testing whether mitoSTAT3-associated ETC control indirectly supports DHODH-dependent mitochondrial ferroptosis defense (Liu Y. et al., 2023; Lahiri et al., 2021; Lee and Roh, 2025).

Within this context, the mitoSTAT3-DHODH framework is best interpreted as a compartment-resolved synthesis rather than a claim that a wholly new ferroptosis pathway has been established. The experimentally supported components include DHODH-mediated CoQ reduction and mitochondrial ferroptosis defense. The proposed connection between HPV-biased STAT3 routing, ETC-dependent CoQ cycling, and DHODH-linked CoQH_2_-dependent protection remains mechanistically plausible but requires direct validation in cervical cancer models. Defining this evidence boundary is central to the organization of the present review. The major HPV-driven metabolic and redox pressures relevant to ferroptosis sensitivity in cervical cancer are summarized in Table 1.

**TABLE 1:** HPV-driven metabolic and redox pressures relevant to ferroptosis sensitivity in cervical cancer.

HPV-related driver	Main biological effect	Ferroptosis-relevant consequence	Evidence status
E6-driven metabolic rewiring	Supports growth signaling, PPP activity, NADPH generation, and cytosolic GSH regeneration	May strengthen GPX4-dependent antioxidant protection while increasing metabolic redox demand	Supported in HPV/cervical cancer models (Pal and Kundu, 2019; Li and Sui, 2021; Pappa et al., 2021; Ortiz-Pedraza et al., 2023; Gore et al., 2025; Tsoneva and Yordanov, 2025; Heber et al., 2023; Chandel et al., 2020; Meng et al., 2024; Chang et al., 2021; Tian et al., 2024)
E7-driven replication stress	Promotes S-phase entry, replication stress, nucleotide demand, and mitochondrial biosynthetic support	Increases dependence on mitochondrial respiration and redox stability	Supported by HPV biology and replication-stress studies (Wendel et al., 2022; Lindemann et al., 2021; Cararo-Lopes et al., 2021; Zhao et al., 2025; Kirschberg et al., 2020)
Glutamine and biosynthetic dependence	Supports anaplerosis and macromolecule synthesis	May increase mitochondrial electron input and oxidative pressure	Supported; ferroptosis linkage is inferential (Ortiz-Pedraza et al., 2023; Gore et al., 2025; Tsoneva and Yordanov, 2025; Tian et al., 2024)
CCRT-induced ROS surge	Adds exogenous oxidative and membrane stress	May expose compartment-specific ferroptosis vulnerability	Clinically relevant; mechanistic validation needed (Chang and Miao, 2023; Zhang et al., 2023a; Bhardwaj and He, 2020; Wang et al., 2022)
Mitochondrial redox adaptation	Preserves ETC function and limits electron leakage	May increase tolerance to mitochondrial LPO	Plausible and testable in this context (Liu et al., 2023a; Lahiri et al., 2021; Tsoneva and Yordanov, 2025)

## 3 HPV-driven metabolic pressure and redox vulnerability: setting the stage for ferroptosis sensitivity

HPV-positive cervical cancer is shaped by increased biosynthetic demand, replication stress, and oxidative burden driven by E6 and E7 (Chang and Miao, 2023; Ortiz-Pedraza et al., 2023; Gore et al., 2025; Tsoneva and Yordanov, 2025). Together, these changes increase the need for redox control during tumor survival (Pappa et al., 2021; Gore et al., 2025; Tsoneva and Yordanov, 2025). In this section, we outline how E6-driven metabolic rewiring and E7-driven replication stress together elevate mitochondrial demand, reshape compartment-specific redox vulnerability, and set the stage for ferroptosis sensitivity (Chang and Miao, 2023; Li et al., 2025b; Ortiz-Pedraza et al., 2023; Gore et al., 2025; Tsoneva and Yordanov, 2025).

### 3.1 E6-driven biosynthetic and redox supply: sustaining growth under constraint

HPV-positive cells proliferate even when the microenvironment is limited (Gore et al., 2025; Heber et al., 2023; Chandel et al., 2020). E6 contributes to this capacity by sustaining growth and survival signaling (Pal and Kundu, 2019; Gore et al., 2025). HPV16 E6 has been reported to sustain receptor tyrosine kinase inputs and preserve mechanistic target of rapamycin (mTOR) activity under growth factor deprivation or nutrient stress (Pal and Kundu, 2019; Chandel et al., 2020). This sustained mTOR activity extends beyond proliferative licensing and contributes to the reprogramming of carbon utilization and electron handling (Li and Sui, 2021; Gore et al., 2025). This accelerated carbon metabolism carries a redox cost. Higher metabolic flux can increase mitochondrial electron input, electron leakage, and ROS generation (Tsoneva and Yordanov, 2025).

To meet this increased redox demand and limit LPO, E6 rewires host metabolism to secure reducing equivalents (Gore et al., 2025; Meng et al., 2024). The primary mechanism is the upregulation of glucose-6-phosphate dehydrogenase, the rate-limiting enzyme of the pentose phosphate pathway (PPP) (Pappa et al., 2021; Meng et al., 2024; Chang et al., 2021). Amplified PPP flux maximizes NADPH production, which supports cytosolic antioxidant capacity (Pappa et al., 2021; Meng et al., 2024). This NADPH pool is essential for regenerating reduced GSH, which fuels GPX4-dependent detoxification of lipid hydroperoxides (Li and Sui, 2021; Pappa et al., 2021). Thus, E6-driven metabolic reprogramming is not merely a growth strategy but also contributes directly to redox protection (Gore et al., 2025; Meng et al., 2024).

HPV oncoproteins can also shift nutrient use toward glutamine metabolism (Gore et al., 2025; Meng et al., 2024). Mechanistic profiling indicated that HPV16 E6/E7 expression increased glutamine transporters and metabolic enzymes that support anaplerotic and macromolecule biosynthesis (Ortiz-Pedraza et al., 2023). Greater dependence on glutamine metabolism can increase mitochondrial electron input and oxidative pressure (Tsoneva and Yordanov, 2025; Tian et al., 2024). When respiratory control is insufficient, this increased electron flux may enhance electron leakage and exacerbate mitochondrial stress (Tsoneva and Yordanov, 2025).

Taken together, E6 helps sustain cytosolic redox protection. It supports growth while maintaining the reducing power needed to restrain LPO (Li and Sui, 2021; Meng et al., 2024). This protective adaptation creates a sustained oxidative burden, part of which may be shifted toward mitochondria, thereby increasing local vulnerability to ferroptotic injury (Tsoneva and Yordanov, 2025).

### 3.2 E7-driven replication stress: rising mitochondrial demand and ROS burden

If E6 supplies the fuel, E7 presses the accelerator (Wendel et al., 2022). It disables the retinoblastoma protein brake and enforces S-phase entry (Wendel et al., 2022; Lindemann et al., 2021). This results in increased deoxyribonucleic acid (DNA) synthesis, more replication conflicts, and sustained demand for nucleotides (Lindemann et al., 2021; Cararo-Lopes et al., 2021). DNA replication is an energetically and biosynthetically costly process (Zhao et al., 2025). To sustain rapid DNA replication, cells must expand their nucleotide pools while preserving biosynthetic and redox balance, thereby increasing their reliance on mitochondrial metabolism (Tsoneva and Yordanov, 2025; Zhao et al., 2025). Mitochondria support aspartate availability and redox handling, both of which are required for efficient nucleotide biosynthesis (Zhao et al., 2025). By driving cells into a replication-intensive state, E7 increases their baseline dependence on mitochondrial function and respiratory stability (Tsoneva and Yordanov, 2025; Kirschberg et al., 2020).

Replication pressure can amplify oxidative stress (Cararo-Lopes et al., 2021; Hochmann et al., 2023). Fork stalling and DNA damage signaling can activate ROS-producing pathways (Lindemann et al., 2021), whereas continued high biosynthetic flux further increases cellular redox demand (Hochmann et al., 2023). This creates a dual-compartment redox challenge: cytosolic demand for NADPH/GSH and mitochondrial imperative for stable respiration with minimal electron leak (Tsoneva and Yordanov, 2025). High-risk E7 is a prominent substrate for casein kinase 2 (CK2), a phosphorylation event critical for its oncogenic persistence (Dizanzo et al., 2022). More importantly, for redox adaptation, this E7-driven kinase rewiring fosters a permissive signaling environment that favors stress-responsive cascades, a feature that primes the cell for the targeted subcellular routing of survival factors, such as STAT3 (Valle‐Mendiola et al., 2023; Gutiérrez‐Hoya and Soto‐Cruz, 2020).

Taken together, E7 imposes a dual mandate: it supports replication while increasing mitochondrial demand and oxidative stress (Tsoneva and Yordanov, 2025; Cararo-Lopes et al., 2021). When therapy induces an exogenous ROS surge, this E7-driven baseline ultimately dictates the margin between adaptation and collapse (Chang and Miao, 2023; Wendel et al., 2022; Ramesh et al., 2023).

### 3.3 Oxidative stress as selection: compartmental vulnerability and the STAT3 routing question

HPV-positive cervical cancer develops under sustained metabolic pressure and elevated ROS, and CCRT imposes an additional oxidative burden (Li and Sui, 2021; Pappa et al., 2021; Tsoneva and Yordanov, 2025; Rashmi et al., 2018). Surviving tumor cells may reflect both favorable genetic features and sufficient redox-adaptive capacity to withstand oxidative stress (Pappa et al., 2021; Rashmi et al., 2018; Calaf et al., 2018). Since ferroptosis is driven by membrane-localized LPO, whole-cell antioxidant measurements may not fully reflect compartment-specific ferroptotic damage (Chang and Miao, 2023; Li et al., 2025b). Cytosolic antioxidant defenses may remain functional while lipid damage accumulates in specific membrane compartments (Mello et al., 2015; Noctor and Foyer, 2016).

E6-associated metabolic rewiring supports the cytosolic NADPH–GSH–GPX4 defense axis (Chang and Miao, 2023; Pappa et al., 2021; Meng et al., 2024). The IMM is lipid-rich and directly exposed to electron flow through the ETC (Liu Y. et al., 2023; Cruz‐Gregorio et al., 2019). When electron transfer becomes inefficient, ROS leakage increases locally, and LPO may propagate within this compartment (Chang and Miao, 2023; Cruz‐Gregorio et al., 2019). In HPV-positive cervical cancer, mitochondria may define the upper limit of redox tolerance (Tsoneva and Yordanov, 2025). Thus, mitochondrial failure may trigger ferroptotic collapse even when cytosolic redox defenses remain functional (Liu Y. et al., 2023).

This mitochondrial vulnerability highlights STAT3 because STAT3 is linked to survival signaling and mitochondria-associated redox control (Liu Y. et al., 2023; Strobel et al., 2023; Zhang et al., 2025). Although STAT3 is widely recognized for its nuclear role downstream of HPV-associated inflammatory and growth signaling, a mitochondria-associated STAT3 fraction has also been described (Liu Y. et al., 2023). Current evidence suggests that chronic HPV-driven metabolic pressure may favor the engagement of mitoSTAT3, potentially linking STAT3 routing to mitochondrial redox control (Liu Y. et al., 2023; Zhang et al., 2025). The following sections examine how this possibility may intersect with DHODH-dependent mitochondrial ferroptosis defense (Liu Y. et al., 2023).

## 4 STAT3 signaling in HPV-positive cervical cancer: activation is not enough - routing is the point

STAT3 is commonly recognized as a canonical oncogenic transcription factor in HPV-positive cervical cancer (Strobel et al., 2023). However, its role may extend beyond nuclear transcription under chronic oxidative stress (Liu Y. et al., 2023; Strobel et al., 2023; Zhang et al., 2025). Nuclear STAT3 orchestrates survival and immune evasion transcriptional programs, and mitochondria-associated STAT3 may modulate bioenergetic stress handling and ROS leakage (Liu Y. et al., 2023; Strobel et al., 2023; Lahiri et al., 2021). This distinction becomes particularly important when therapy amplifies oxidative stress and promotes LPO-mediated membrane injury (Chang and Miao, 2023; Yong et al., 2024; Cruz‐Gregorio et al., 2023).

### 4.1 Structural logic and phosphorylation control: Tyr705 nuclear signaling vs. Ser727 stress outputs

STAT3 has both canonical transcriptional and non-canonical mitochondrial functions (Hu et al., 2024; Arévalo et al., 2023). In the canonical pathway, STAT3 translocates to the nucleus and regulates transcription. In parallel, a smaller mitochondria-associated fraction has been reported to influence mitochondrial metabolism and redox control (Adamo et al., 2021; Mohammed et al., 2020). This partitioning is closely related to STAT3 phosphorylation status and domain structure (Berkley et al., 2025). The Src homology 2 (SH2) domain serves as a critical dimerization hub, facilitating the Tyr705-dependent assembly required for canonical nuclear signaling (Hu et al., 2024; Arévalo et al., 2023). The C-terminal transactivation domain harbors Ser727, a residue specifically targeted by stress-adaptive kinase cascades (Arévalo et al., 2023). Tyr705 phosphorylation initiates this canonical program (Berkley et al., 2025). Upstream kinases, particularly Janus kinase (JAK), execute this modification (Berkley et al., 2025). Tyr705-phosphorylated STAT3 rapidly dimerizes through its SH2 interface and translocates to the nucleus, effectively translating extracellular signals into transcription (Hu et al., 2024; Arévalo et al., 2023; Johnson et al., 2018; Johnston and Grandis, 2011; Hua et al., 2022).

Ser727 phosphorylation carries a different functional meaning. It is primarily implicated in non-canonical effector mechanisms (Stenckova et al., 2022). Ser727 phosphorylation imparts a distinct functional signature associated with mitoSTAT3 activity and manifests as changes in respiratory efficiency and ROS generation (Arévalo et al., 2023; Mohammed et al., 2020). However, this does not imply that Ser727 is a universal “mitochondrial zip code,” as recent structural evidence indicates that mitoSTAT3 targeting can depend on internal sequence motifs rather than Ser727 phosphorylation alone (Marié et al., 2024). Instead, the Tyr705–Ser727 balance may help determine whether STAT3 activity is directed mainly toward nuclear transcription or mitochondria-associated stress control (Stenckova et al., 2022).

### 4.2 How HPV sustains STAT3 and directs its output: interleukin-6 (IL-6) loops, kinase wiring, and Ser727 signaling

Sustained STAT3 activation has been observed in HPV-positive cervical cancer models (Strobel et al., 2023; Valle‐Mendiola et al., 2023; Gutiérrez‐Hoya and Soto‐Cruz, 2020; Scarth et al., 2021). Oxidative stress and kinase pathway activation have also been associated with increased STAT3 Ser727 phosphorylation in diverse systems (Mohammed et al., 2020). However, whether Ser727 phosphorylation directly dictates mitoSTAT3 localization remains uncertain (Mohammed et al., 2020). Malignancies driven by high-risk HPV rarely rely on an isolated linear signaling pathway. Instead, they maintain overlapping signaling circuits that support survival under chronic stress (Chang and Miao, 2023). A major contributor to sustained STAT3 activity is the IL-6–mediated autocrine loop (Scarth et al., 2021; Rossetti et al., 2020; Pagni et al., 2022). HPV-associated tumors frequently foster inflammatory amplification within the tumor microenvironment (Rossetti et al., 2020). IL-6 receptor stimulation activates JAK kinases, preserves Tyr705 phosphorylation, and enforces canonical nuclear signaling (Valle‐Mendiola et al., 2023; Gutiérrez‐Hoya and Soto‐Cruz, 2020). These downstream signals reinforce apoptosis resistance and immune evasion through checkpoint-ligand upregulation and cytokine remodeling (Strobel et al., 2023).

HPV oncoproteins may reinforce this persistence. By weakening p53-dependent safeguards, E6 supports survival signaling and allows stressed cells to persist rather than undergo elimination (Zhang et al., 2025; Scarth et al., 2021). This chronic pressure may convert STAT3 from a transient stress responder into a mediator of longer-term adaptation (Zhang et al., 2025). E7 employs a distinct strategy. In addition to disrupting the Rb/E2F transcription factor checkpoint axis, E7 reshapes the cellular signaling milieu in ways that may favor kinase activity patterns relevant to STAT3 regulation (Valle‐Mendiola et al., 2023; Gutiérrez‐Hoya and Soto‐Cruz, 2020; Morgan and Macdonald, 2020). In response to sustained oxidative stress, a fraction of STAT3 may become Ser727-modified and show increased mitochondrial localization (Mohammed et al., 2020). This framework may explain how HPV-positive tumors maintain a mitochondria-associated STAT3 fraction under chronic stress (Mohammed et al., 2020).

It is instructive to distinguish between established facts and mechanistic assumptions. Sustained STAT3 activation in HPV-positive disease is well documented (Strobel et al., 2023; Scarth et al., 2021). However, it remains unclear whether HPV-associated kinase signaling directly routes STAT3 to mitochondria (Mohammed et al., 2020). This distinction leads to a testable question: if CK2 or related stress kinases contribute to Ser727-linked mitochondrial STAT3 signaling, their inhibition may reduce mitochondrial redox tolerance, especially during therapy-induced oxidative stress (Mohammed et al., 2020; Rosales et al., 2022). Targeting STAT3 in advanced disease is challenging because therapy itself may reinforce the same inflammatory signals that sustain this pathway (Strobel et al., 2023). CCRT extends beyond DNA damage and reshapes the cytokine milieu (Chang and Miao, 2023). CCRT-induced inflammation may increase IL-6 signaling, which can reactivate the JAK–STAT3 pathway and sustain Tyr705-dependent nuclear STAT3 activity (Rossetti et al., 2020).

In parallel, oxidative stress can intensify the PI3K/Akt/mTOR signaling pathway (Valle‐Mendiola et al., 2023). Under sustained kinase activation, STAT3 may acquire both Tyr705-and Ser727-linked signals rather than being restricted to a single output (Valle‐Mendiola et al., 2023; Mohammed et al., 2020; Scarth et al., 2021). Cells do not necessarily select between nuclear and mitochondrial pools; rather, they may engage both (Mohammed et al., 2020). Under chronic ROS stress, this coexistence makes mitochondrial routing consequential: Ser727-linked outputs may stabilize the mitochondrial redox balance and increase resistance to inner-membrane failure (Mohammed et al., 2020). In this context, mitoSTAT3 may represent a potentially relevant mitochondrial vulnerability that warrants further investigation (Gutiérrez‐Hoya and Soto‐Cruz, 2020).

### 4.3 MitoSTAT3: ETC efficiency, ROS leak, and implications for CoQ redox cycling

The mitoSTAT3 fraction has been reported in diverse biological contexts (Conway et al., 2022; Comità et al., 2021; Yang and Rincon, 2016). Rather than serving merely as a structural component, this pool appears to be functionally responsive to cellular stress, with recent evidence identifying a STAT3-containing mitochondrial protein complex involved in mitochondrial mRNA stability and cancer-related mitochondrial function (Conway et al., 2022; Mohammed et al., 2020; Gil et al., 2026). The precise mechanisms of mitochondrial import remain under investigation, heat shock protein 90, GRIM-19-associated processes, and STAT3 sequence features have been implicated in different systems (Marié et al., 2024; Comità et al., 2021; Yang and Rincon, 2016; Zouein et al., 2015). Current evidence supports that a distinct fraction of STAT3 can localize to mitochondria and is often associated with Ser727-linked stress signaling (Conway et al., 2022; Mohammed et al., 2020; Marié et al., 2024; Comità et al., 2021; Yang and Rincon, 2016; Zouein et al., 2015).

MitoSTAT3 is considered to be a regulator of mitochondrial bioenergetic efficiency (Lahiri et al., 2021; Yang and Rincon, 2016; Brambilla et al., 2020). Many studies have linked mitoSTAT3 to Complex I-associated behavior, whereas other reports have inferred its effects from changes in oxygen consumption, coupling efficiency, or membrane potential stability (Lahiri et al., 2021; Comità et al., 2021; Yang and Rincon, 2016; Xu et al., 2016). Across these studies, a recurring pattern has emerged: mitoSTAT3 is frequently associated with more efficient electron flux and reduced electron leak, particularly under oxidative or metabolic stress rather than under basal conditions (Mohammed et al., 2020; Yang and Rincon, 2016). This distinction is important because electron leak is not simply a generic source of oxidant burden. When electrons escape from the ETC, superoxide and related reactive species increase locally, and the IMM is positioned closest to this source (Liu Y. et al., 2023; Waypa et al., 2016). Under sustained stress, lipid-radical chain reactions may therefore propagate within the same compartment in which electron leakage occurs (Liu Y. et al., 2023). In this way, mitochondrial membranes become a key site at which ferroptotic vulnerability may become rate-limiting rather than being diluted across the cell as a whole (Chang and Miao, 2023; Liu Y. et al., 2023).

ETC activity also shapes the redox state of the CoQ pool (Liu Y. et al., 2023), and this balance influences whether the local radical-trapping capacity can be regenerated within the inner membrane (Mishima et al., 2023; Wang and Min, 2021). DHODH is particularly relevant in this context because it contributes to CoQH_2_ generation, thereby supporting local protection against lipid radical propagation (Liu Y. et al., 2023; Lee and Roh, 2025; Zeng et al., 2022; Li et al., 2025c). This function depends on the continuous regeneration of oxidized CoQ through ETC-supported redox cycling (Liu Y. et al., 2023). If ETC performance deteriorates and CoQ turnover becomes inefficient, DHODH-dependent membrane protection may be constrained, even in the presence of DHODH (Mishima et al., 2023; Wang and Min, 2021).

Taken together, mitoSTAT3 may provide a functional link between mitochondrial redox control and ferroptosis defense (Chang and Miao, 2023; Liu Y. et al., 2023). By supporting ETC efficiency and limiting electron leakage, it may help preserve the CoQ redox environment required for DHODH-linked membrane protection (Liu Y. et al., 2023; Mohammed et al., 2020; Yang and Rincon, 2016). The protective role of the DHODH–CoQ axis is increasingly well supported, whereas the proposition that mitoSTAT3 actively sustains this axis through ETC/CoQ tuning is better framed as a testable working model (Liu Y. et al., 2023; Lee and Roh, 2025; Wang and Min, 2021; Zeng et al., 2022). Even so, this framework offers a coherent explanation for how STAT3 routing could influence mitochondrial ferroptosis sensitivity in HPV-positive cervical cancer (Chang and Miao, 2023; Wang et al., 2023; Strobel et al., 2023; Zhang et al., 2025).

### 4.4 Therapeutic persistence and pathway crosstalk: why STAT3 signaling rebounds under CCRT

Suppressing STAT3 in advanced cervical cancer is difficult because it is embedded in a redundant, self-reinforcing circuit (Strobel et al., 2023; Xing et al., 2023; Liang et al., 2024). When one upstream input is blocked, parallel pathways compensate, and under therapeutic stress, this compensation often strengthens rather than fading (Yang et al., 2020). Cisplatin and radiation trigger a local inflammatory surge, which is often rich in IL-6 (Rossetti et al., 2020; Liang et al., 2024). This response can sustain or reactivate nuclear factor κB–STAT3 feed-forward signaling during and after chemoradiotherapy (Rossetti et al., 2020; Xing et al., 2023).

Two reinforcing circuits are particularly important in this regard. The PI3K/Akt/mTOR axis promotes cell survival and increases resistance to oxidative stress (Cruz‐Gregorio and Aranda‐Rivera, 2021; Liu et al., 2022). Beyond metabolic adaptation, it intersects with cytokine programs that keep STAT3 activated (Liu et al., 2022). Under therapy-induced DNA damage, cells frequently recruit the PI3K/Akt pathway as a compensatory survival route (Götting et al., 2020; Alemi et al., 2021; Karimian et al., 2018). Once engaged, STAT3 can be maintained indirectly through convergent upstream effectors and transcriptional programs that preserve the sensitivity of the receptors (Morgan and Macdonald, 2019a; Noh et al., 2022). NF-κB also provides an additional inflammatory reinforcement pathway. Its activation promotes IL-6 secretion and broader cytokine outputs, stabilizing the autocrine milieu required to maintain the Tyr705-associated STAT3 signaling (Rossetti et al., 2020; Xing et al., 2023). In this setting, therapy-induced inflammation can carry a trade-off: it may facilitate rebound signaling and help tumors regain momentum after the initial cytotoxic insult (Liang et al., 2024).

These feedback loops also shape subcellular routing. Persistent cytokine and kinase inputs may favor the coexistence of multiple STAT3 phosphorylation states (Yang and Rincon, 2016; Morgan et al., 2018; Morgan and Macdonald, 2019b). In this setting, STAT3 is not confined to a single compartmental pool (Yang and Rincon, 2016; Avalle and Poli, 2018). Nuclear and mitochondria-associated outputs can coexist, and their relative contributions are context dependent (Lahiri et al., 2021). Under chronic oxidative stress, non-canonical mitochondrial outputs are important because they may help define the boundary between mitochondrial adaptation and redox failure (Mohammed et al., 2020).

This perspective shifts the framing of STAT3 in the remainder of this review. STAT3 is therefore considered not only a transcription factor but also a stress-adaptive signaling mediator. This logic also supports the mitochondrial mechanisms. Therapy amplifies ROS and LPO pressure, and pathways that preserve mitochondrial redox stability emerge as key determinants of survival (Liu Y. et al., 2023; Ma et al., 2025; Kleih et al., 2019; Pearson et al., 2021). In this framework, the mitoSTAT3–ETC/CoQ–DHODH interface is no longer a peripheral observation but a coherent and mechanistically actionable target (Chang and Miao, 2023; Li et al., 2025b; Yong et al., 2024). Table 2 summarizes the main STAT3 signaling outputs and their ferroptosis-relevant implications in HPV-positive cervical cancer.

**TABLE 2:** STAT3 signaling outputs and ferroptosis-relevant implications in HPV-positive cervical cancer.

STAT3 feature	Main output	Ferroptosis-relevant implication	Evidence status
Tyr705-associated nuclear STAT3	Transcriptional survival, immune evasion, and inflammatory programs	May indirectly sustain resistance to oxidative stress and therapy	Established in cancer; context supported in HPV-positive disease (Strobel et al., 2023; Valle‐Mendiola et al., 2023; Gutiérrez‐Hoya and Soto‐Cruz, 2020; Hu et al., 2024; Arévalo et al., 2023; Adamo et al., 2021; Mohammed et al., 2020; Berkley et al., 2025; Johnson et al., 2018; Johnston and Grandis, 2011; Hua et al., 2022)
Ser727-associated stress signaling	Non-canonical stress outputs and possible mitochondrial enrichment	May support mitochondrial redox adaptation	Supported across systems; Ser727 phosphorylation alone has not been shown to be sufficient (Arévalo et al., 2023; Mohammed et al., 2020; Stenckova et al., 2022)
mitoSTAT3 fraction	Modulation of ETC behavior, membrane potential, and mitochondrial ROS production	May influence local oxidative stress at ferroptosis-sensitive membranes	Supported in several systems; limited cervical cancer-specific data (Conway et al., 2022; Lahiri et al., 2021; Mohammed et al., 2020; Marié et al., 2024; Gil et al., 2026; Comità et al., 2021; Yang and Rincon, 2016; Zouein et al., 2015; Brambilla et al., 2020; Xu et al., 2016)
IL-6/JAK feedback	Maintains persistent STAT3 activation under inflammation	May reinforce therapy persistence and redox vulnerability	Supported in cervical cancer and inflammatory contexts (Valle‐Mendiola et al., 2023; Gutiérrez‐Hoya and Soto‐Cruz, 2020; Scarth et al., 2021; Rossetti et al., 2020; Pagni et al., 2022; Xing et al., 2023; Liang et al., 2024)
PI3K/Akt/mTOR crosstalk	Compensatory survival and metabolic signaling	May increase mixed STAT3 modification states under stress	Supported; mitochondrial routing remains inferential (Valle‐Mendiola et al., 2023; Mohammed et al., 2020; Cruz‐Gregorio and Aranda‐Rivera, 2021; Liu et al., 2022; Götting et al., 2020; Alemi et al., 2021; Karimian et al., 2018; Morgan and Macdonald, 2019a; Noh et al., 2022)

## 5 DHODH and mitochondrial ferroptosis defense: the mitoSTAT3-ETC-CoQ interface

The framework discussed in this section should be interpreted as mechanistically plausible and testable, rather than fully established. We synthesized compartment-resolved ferroptosis defense with DHODH–CoQ biochemistry. This synthesis generates experimentally tractable predictions about how HPV-biased STAT3 routing might influence ETC-dependent CoQ cycling and mitochondrial protection against LPO.

HPV-positive cervical cancer evolves under chronic oxidative pressure (Chang and Miao, 2023; Li et al., 2025b). Basal ROS levels are elevated, and CCRT imposes a second surge (Chang and Miao, 2023; Li et al., 2025b; Tsoneva et al., 2024). This context has clinical significance: cisplatin-based CCRT and radiation exacerbate membrane injury (Wang et al., 2022), selecting for survivors with robust redox defenses (Liu Y. et al., 2023). Under this selection, ferroptosis is not merely conceptual; it becomes a tangible metabolic vulnerability when LPO concentrates within specific compartments (Chang and Miao, 2023; Wang et al., 2022).

Therefore, it is useful to adopt a more compartment-resolved view of ‘antioxidant defense’ rather than treating it as a single, uniform category (Liu Y. et al., 2023; Zhang X. et al., 2023). Cells defend membranes with distinct architectures; some systems act broadly, while others are confined to specific membrane compartments (Liu Y. et al., 2023). For instance, while ferroptosis suppressor protein one serves as a dedicated guardian of the plasma membrane, it is spatially excluded from the mitochondrial matrix (Liu Y. et al., 2023; Doll et al., 2019; Chen Z. et al., 2023). Here, we focus on the mitochondrial layer. We trace the path from compartmentalized defense to DHODH-CoQ biochemistry and then to a coupling model that links HPV-biased STAT3 routing to mitochondrial ferroptosis defense (Li et al., 2025b; Strobel et al., 2023; Lee and Roh, 2025; Zhang et al., 2025; Wang and Min, 2021). Conceptually, we propose that HPV-driven stress may bias STAT3 toward Ser727-linked mitochondrial outputs (Mohammed et al., 2020). In turn, this shift could help stabilize ETC-dependent CoQ redox cycling and thereby support DHODH-linked CoQH_2_ protection, reducing susceptibility to mitochondrial ferroptotic injury under oxidative pressure (Liu Y. et al., 2023; Lahiri et al., 2021; Brambilla et al., 2020; Mishima et al., 2023).

### 5.1 Spatial organization of ferroptosis defense: why membranes matter

Ferroptosis is fundamentally a membrane-centered form of oxidative injury (Liu Y. et al., 2023). Lipid radicals originate and propagate within hydrophobic bilayers, suggesting that this self-amplifying chain reaction requires radical-trapping antioxidants precisely at the site of initiation (Wang and Min, 2021). For this reason, ferroptosis defense is inherently compartment-specific rather than uniformly distributed across the cell (Liu Y. et al., 2023; Gopal et al., 2023). This spatial logic helps explain why distinct ferroptosis-defense modules are better understood as complementary rather than redundant (Chen Z. et al., 2023). The canonical GPX4–GSH axis acts broadly by reducing phospholipid hydroperoxides across multiple membrane pools and therefore provides a major baseline defense against ferroptotic damage (Liu Y. et al., 2023; Chen Z. et al., 2023). The plasma membrane FSP1 pathway represents a second layer, regenerating reduced CoQ at the cell surface to support local radical-trapping activity (Liu Y. et al., 2023; Doll et al., 2019; Chen Z. et al., 2023). A third defense layer is localized to mitochondria and is mediated in part by DHODH. By linking metabolism to the mitochondrial CoQ pool, DHODH may help sustain local CoQH_2_ availability and thereby limit lipid-radical propagation within the IMM (Liu Y. et al., 2023; Wang and Min, 2021).

This spatial organization is highly relevant to HPV-positive tumors, which frequently operate near the limits of their mitochondrial redox capacity (Chang and Miao, 2023; Li et al., 2025b). Consequently, the inner membrane CoQ pool and its ETC-dependent turnover may become a major determinant of whether oxidative stress remains tolerable or progresses toward ferroptotic collapse (Gopal et al., 2023). From this perspective, the critical question is not which tumors express more antioxidant genes, but which membrane compartment bears the dominant oxidative burden and which defense layer carries the greatest functional load. This compartmental reliance is further enforced by upstream metabolic constraints. System Xc^−^ and cysteine availability define the upper bound for GSH synthesis and, therefore, influence GPX4-dependent protection (Chang and Miao, 2023; Liu Y. et al., 2023; Koppula et al., 2021). Under chronic ROS stress, cytosolic redox defenses may become strained, increasing reliance on CoQ-based backup systems (Liu Y. et al., 2023; Lv et al., 2023). In this context, HPV-positive tumors may not only benefit from mitochondrial protection but may also become functionally dependent on it (Chang and Miao, 2023; Wang et al., 2022; Li et al., 2025b). This possibility is especially important because it can be tested experimentally. For instance, perturbation of DHODH/CoQ-linked protection under therapy-mimetic oxidative stress may reveal its contribution to mitochondrial LPO (Liu Y. et al., 2023; Wang and Min, 2021; Ma et al., 2022).

### 5.2 DHODH-CoQ biochemistry: CoQH_2_-dependent protection in the inner membrane

DHODH is an IMM-anchored flavoprotein that transfers electrons from dihydroorotate to the mitochondrial CoQ pool (Liu Y. et al., 2023; Lee and Roh, 2025; Rodriguez et al., 2022a). Oxidized CoQ serves as the electron acceptor for DHODH, whereas CoQH_2_ provides radical-trapping activity that protects the membranes (Mishima et al., 2023; Zeng et al., 2022; Tang et al., 2021). Thus, protection depends on dynamic CoQ redox cycling, not simply on the oxidized CoQ pool (Liu Y. et al., 2023). This positioning couples pyrimidine metabolism to the CoQ pool, making DHODH flux dependent on ETC-supported CoQ redox turnover that regenerates oxidized CoQ and thereby permits sustained CoQH_2_ production (Lee and Roh, 2025; Mishima et al., 2023). While topological models vary, the exact orientation is subordinate to the interface itself: DHODH bridges a metabolic substrate and a lipid-soluble redox pool, generating local CoQH_2_ precisely where protection is essential (Rodriguez et al., 2022a; Rodriguez et al., 2022b).

CoQH_2_ is important because it can interrupt lipid-radical chain reactions within membranes (Zeng et al., 2022; Tang et al., 2021). In the IMM, lipid-radical formation may occur near sites of ETC-derived ROS leakage, making local CoQH_2_-dependent protection especially relevant (Liu Y. et al., 2023; Mishima et al., 2023). DHODH contributes to this protective CoQH_2_ pool by reducing CoQ during pyrimidine metabolism (Lee and Roh, 2025; Mishima et al., 2023). However, DHODH is not the only pathway that influences CoQH_2_ availability. Other CoQ-reducing systems and the downstream activity of the ETC also shape the balance between oxidized CoQ and reduced CoQH_2_ (Liu Y. et al., 2023; Gopal et al., 2023). Therefore, DHODH-dependent protection should be understood as part of a dynamic CoQ redox cycle rather than as a fixed, standalone antioxidant system.

Recent evidence supports a distinct role for DHODH in mitochondrial ferroptosis defense (Lee and Roh, 2025; Mishima et al., 2023; Wang and Min, 2021). Rather than replacing GPX4, DHODH provides a compartment-specific mitochondrial protection mechanism (Liu Y. et al., 2023; Wang and Min, 2021; Ma et al., 2022; Zeng et al., 2024). Whether HPV-positive cervical cancer exhibits heightened dependency on this mitochondrial defense layer remains to be validated (Chang and Miao, 2023). This spatial complementarity becomes evident when DHODH inhibition triggers lethal mitochondrial LPO even when GPX4 function remains intact (Liu Y. et al., 2023; Mishima et al., 2023; Ma et al., 2022). This highlights that ferroptotic cell death can be driven by a localized breakdown in mitochondrial defense rather than global antioxidant collapse (Wang and Min, 2021; Zeng et al., 2024).

### 5.3 ETC-driven CoQ redox cycling: a prerequisite for DHODH-linked membrane protection

DHODH transfers electrons from dihydroorotate to oxidized CoQ, producing reduced CoQH_2_ (Liu Y. et al., 2023; Mishima et al., 2023; Wang and Hekimi, 2021). The CoQ pool continuously cycles between oxidized CoQ and reduced CoQH_2_ (Pallotti et al., 2021). Therefore, DHODH activity depends not only on DHODH abundance but also on the availability of oxidized CoQ (Gopal et al., 2023; Hubáčková et al., 2020). This coupling links pyrimidine metabolism to mitochondrial respiration via continuous CoQ redox cycling (Lee and Roh, 2025; Zhou et al., 2021). DHODH reduces CoQ to CoQH_2_, thereby contributing to localized radical-trapping capacity within the IMM (Liu Y. et al., 2023; Wang and Min, 2021). The ETC then reoxidizes CoQH_2_ back to CoQ as electrons pass through the respiratory chain to oxygen (Mishima et al., 2023; Gopal et al., 2023). Thus, DHODH-dependent membrane protection relies on ongoing CoQ turnover rather than the simple presence of CoQ (Liu Y. et al., 2023; Wang and Hekimi, 2021).

When electron flow through the ETC becomes inefficient, the balance between oxidized CoQ and reduced CoQH_2_ may become disturbed (Wang and Hekimi, 2021). One possible consequence is an excessively reduced CoQ pool: if CoQH_2_ accumulates without efficient re-oxidation, DHODH loses access to oxidized CoQ, and its flux becomes constrained (Mishima et al., 2023; Hubáčková et al., 2020). At the same time, ETC inefficiency can increase electron leakage and local ROS, promoting LPO in the IMM (Liu Y. et al., 2023; Gopal et al., 2023). Conversely, if upstream electron entry is limited, the pool may become excessively oxidized, reducing the local availability of protective CoQH_2_ (Pallotti et al., 2021). These two extremes differ mechanistically, but both indicate that membrane protection depends on dynamic CoQ cycling rather than on either redox state alone (Liu Y. et al., 2023).

The key issue is not ETC activity alone, but whether ETC function maintains CoQ cycling without excessive electron leakage (Lahiri et al., 2021). MitoSTAT3 may help modulate ETC efficiency under stress (Lahiri et al., 2021; Brambilla et al., 2020). If mitoSTAT3 helps sustain electron flux and reduce electron leakage under chronic HPV-associated stress, it could indirectly support DHODH-dependent membrane protection (Lahiri et al., 2021). This possibility can be viewed as a mechanistically plausible and testable model rather than an established regulatory mechanism.

### 5.4 mitoSTAT3 as a regulator of ETC efficiency: implications for CoQ redox balance

STAT3 is usually viewed as a transcription factor (Yang and Rincon, 2016). However, a smaller fraction of STAT3 can also be found in or around mitochondria (Lahiri et al., 2021; Yang and Rincon, 2016). Mechanistically, mitoSTAT3 has been reported to associate with ETC complexes, most often in connection with Complex I (Yang and Rincon, 2016; Ploeger et al., 2020; Nallar and Kalvakolanu, 2016). GRIM-19-associated mechanisms and STAT3 sequence features have been proposed to contribute to mitochondrial STAT3 localization (Marié et al., 2024; Yang and Rincon, 2016; Zouein et al., 2015; Nallar and Kalvakolanu, 2016). The strength of evidence for direct physical binding differs across experimental systems (Marié et al., 2024; Yang and Rincon, 2016; Zouein et al., 2015; Nallar and Kalvakolanu, 2016). We discuss mitoSTAT3 as a regulator of ETC efficiency and mitochondrial ROS production, rather than as a fixed structural component of a specific ETC complex (Lahiri et al., 2021; Yang and Rincon, 2016; Brambilla et al., 2020).

MitoSTAT3 may be relevant in HPV-positive cervical cancer because viral oncogenic signaling is associated with high metabolic flux (Strobel et al., 2023; Piao et al., 2020; Shukla et al., 2019; Allen et al., 2022). HPV oncoproteins promote anabolic processes and replication programs, elevating mitochondrial burden (Chang and Miao, 2023; Cruz‐Gregorio et al., 2019; Allen et al., 2022). In this setting, even slight alterations in ETC efficiency may result in significant consequences for electron leakage and membrane stability (Lahiri et al., 2021; Brambilla et al., 2020). CoQ turnover is required for both respiration and CoQH_2_-mediated radical trapping (Liu Y. et al., 2023; Lee and Roh, 2025). By supporting ETC flux and limiting electron leakage, mitoSTAT3 may help preserve CoQ cycling and thereby indirectly support DHODH-linked ferroptosis defense (Liu Y. et al., 2023; Lahiri et al., 2021; Lee and Roh, 2025; Yang and Rincon, 2016; Mishima et al., 2023).

### 5.5 The mitoSTAT3-ETC-DHODH axis as a context-dependent regulatory network of ferroptosis defense

Current evidence suggests that mitoSTAT3, ETC-dependent CoQ turnover, and DHODH-mediated CoQH_2_-dependent protection may form a mitochondrial defense network in HPV-positive cervical cancer (Chang and Miao, 2023; Liu Y. et al., 2023; Lahiri et al., 2021). However, the strength of evidence differs across this network. DHODH-mediated CoQ reduction and DHODH-dependent mitochondrial ferroptosis defense are experimentally supported (Liu Y. et al., 2023; Lee and Roh, 2025; Mishima et al., 2023), whereas direct causal control of CoQ redox cycling and DHODH-dependent ferroptosis defense by mitoSTAT3 remains a testable working model based on converging but still indirect evidence (Lahiri et al., 2021; Yang and Rincon, 2016). In this framework, mitoSTAT3 may support ETC efficiency and limit electron leakage, thereby helping to maintain a CoQ redox environment compatible with DHODH activity (Lahiri et al., 2021; Brambilla et al., 2020; Gopal et al., 2023; Pallotti et al., 2021). In turn, DHODH may contribute to local CoQH_2_ generation within the IMM, thereby helping to restrain mitochondrial LPO under oxidative stress (Liu Y. et al., 2023; Mishima et al., 2023; Wang and Min, 2021; Zeng et al., 2024).

From a therapeutic perspective, this framework highlights the potentially actionable mitochondrial vulnerability in HPV-positive cervical cancer (Chang and Miao, 2023; Liu Y. et al., 2023). Under conditions of treatment-induced oxidative stress, inhibition of DHODH or disruption of mitoSTAT3-associated mitochondrial adaptation may weaken the defense against membrane LPO, thereby sensitizing tumor cells to oxidative injury (Li et al., 2025b; Yong et al., 2024; Jiang et al., 2023). This mechanistic rationale provides the basis for the therapeutic strategies discussed in the following section. The evidence status of each component of the proposed mitoSTAT3–ETC–CoQ–DHODH framework is summarized in Table 3.

**TABLE 3:** Evidence status of the mitoSTAT3-ETC-CoQ-DHODH ferroptosis-defense framework.

Component or link	Current evidence	Interpretation in this review	Validation needed
DHODH reduces CoQ to CoQH_2_	Biochemically established (Liu et al., 2023a; Lee and Roh, 2025; Mishima et al., 2023; Rodriguez et al., 2022a; Wang and Hekimi, 2021; Pallotti et al., 2021; Hubáčková et al., 2020; Zhou et al., 2021)	Validated component of the framework	Confirm flux under therapy-mimetic stress in cervical cancer
DHODH protects against mitochondrial ferroptosis	Supported in cancer models (Liu et al., 2023a; Lee and Roh, 2025; Mishima et al., 2023; Wang and Min, 2021; Ma et al., 2022; Zeng et al., 2024)	Validated but context-dependent defense layer	Genetic and pharmacologic validation in HPV-positive models
ETC supports CoQ redox turnover	Biochemically established (Liu et al., 2023a; Mishima et al., 2023; Gopal et al., 2023; Wang and Hekimi, 2021; Pallotti et al., 2021; Hubáčková et al., 2020; Zhou et al., 2021)	Required background condition for DHODH flux	Measure CoQ/CoQH_2_ ratios during ETC perturbation
mitoSTAT3 modulates ETC efficiency	Supported across several systems (Lahiri et al., 2021; Mohammed et al., 2020; Marié et al., 2024; Gil et al., 2026; Yang and Rincon, 2016; Zouein et al., 2015; Brambilla et al., 2020)	Mechanistically relevant but not cervical cancer-specific	STAT3 localization, mutant rescue, and respiratory profiling
mitoSTAT3 sustains DHODH-linked ferroptosis defense	No direct causal evidence yet (Liu et al., 2023a; Lahiri et al., 2021; Lee and Roh, 2025; Yang and Rincon, 2016; Brambilla et al., 2020; Mishima et al., 2023; Wang and Min, 2021; Gopal et al., 2023)	Testable working model	Combine STAT3 perturbation, DHODH rescue, CoQ redox analysis, and mitochondrial LPO readouts
HPV-positive dependency on this axis	Plausible from viral stress biology (Chang and Miao, 2023; Li et al., 2025b; Strobel et al., 2023; Ortiz-Pedraza et al., 2023; Gore et al., 2025; Tsoneva and Yordanov, 2025; Zhang et al., 2025)	Context-dependent hypothesis	Compare HPV-positive, HPV-negative, and patient-derived models

## 6 Therapeutic strategies targeting the mitoSTAT3-DHODH ferroptosis-defense axis

The evidence summarized in the preceding sections suggests that HPV-positive cervical cancer may exploit a mitochondria-centered redox adaptation program to survive chronic oxidative stress and resist therapy (Chang and Miao, 2023; Wang et al., 2022; Liu Y. et al., 2023). MitoSTAT3, ETC-dependent CoQ cycling, and DHODH-mediated mitochondrial protection together provide a mechanistically plausible basis for therapeutic intervention (Liu Y. et al., 2023; Lahiri et al., 2021; Lee and Roh, 2025). Although chemoradiotherapy remains the standard treatment, its efficacy is frequently constrained by acquired resistance, tumor recurrence, and nonselective toxicity (Chang and Miao, 2023; Strobel et al., 2023). These limitations highlight the need for more precise, mechanism-based strategies that weaken the mitochondrial ferroptosis defense while preserving normal tissue tolerance. Targeted inhibition of STAT3 and DHODH may offer a new translational direction for HPV-positive cervical cancer.

### 6.1 Why the mitoSTAT3-DHODH axis represents a ferroptosis-directed therapeutic vulnerability

The transition from mechanistic understanding to therapeutic development in HPV-positive cervical cancer requires changes in emphasis. Rather than viewing mitochondrial stress adaptation as a by-product of viral transformation, it may be more useful to regard it as a targetable vulnerability (Chang and Miao, 2023; Cruz‐Gregorio et al., 2019). HPV-positive cervical cancer develops under persistent metabolic, replicative, and oxidative stress driven by viral oncoproteins and is further intensified by cisplatin-based chemoradiotherapy (Chang and Miao, 2023; Cruz‐Gregorio et al., 2019; Allen et al., 2022). In this setting, tumor survival is unlikely to depend solely on generalized antioxidant capacity (Liu Y. et al., 2023; Mishima et al., 2023; Mao et al., 2026). Instead, it may rely on compartment-specific defense systems that preserve mitochondrial integrity and restrain LPO under chronic stress (Liu Y. et al., 2023; Wang and Min, 2021; Zeng et al., 2024).

This framework explains why the mitoSTAT3–DHODH axis may be amenable to therapeutic targeting. Current evidence suggests that mitoSTAT3 may support ETC efficiency, limit electron leakage, and help maintain a CoQ redox environment (Liu Y. et al., 2023; Lahiri et al., 2021; Brambilla et al., 2020). DHODH establishes a spatially restricted ferroptosis-defense mechanism within the IMM (Liu Y. et al., 2023; Lee and Roh, 2025; Wang and Min, 2021). Together, these functions may enable HPV-positive cervical cancer cells to tolerate oxidative stress that would otherwise destabilize mitochondrial membranes (Chang and Miao, 2023; Wang et al., 2022; Liu Y. et al., 2023).

The therapeutic value of the mitoSTAT3–DHODH axis lies in the possibility of weakening a local mitochondrial defense system rather than increasing oxidative damage indiscriminately. Standard CCRT already induces substantial oxidative and membrane stress (Shah et al., 2021; Marinescu et al., 2014; Ebrahimi et al., 2018). Under these conditions, interventions targeting mitoSTAT3-associated bioenergetic support or DHODH-mediated local redox protection may compromise the capacity of tumor cells to contain mitochondrial LPO (Li et al., 2025b; Yong et al., 2024; Jiang et al., 2023). In this sense, the goal is not merely to raise ROS levels but to lower the ability of tumor cells to survive ROS within this highly oxidative subcellular microenvironment (Liu Y. et al., 2023; Jiang et al., 2023).

### 6.2 STAT3-targeting strategies: direct inhibition and pathway-level intervention

STAT3 remains a promising therapeutic target because it integrates survival signaling, inflammatory reinforcement, and immune evasion in cervical cancer (Strobel et al., 2023; Valle‐Mendiola et al., 2023). Existing approaches can be broadly divided into direct inhibition of STAT3 and indirect suppression of upstream pathways that maintain its activity (Valle‐Mendiola et al., 2023). Direct strategies include inhibitors that interfere with STAT3 phosphorylation, dimerization, DNA binding, or transcriptional output, whereas indirect strategies target IL-6/JAK signaling, receptor tyrosine kinases, and related pathway crosstalk (Valle‐Mendiola et al., 2023). In HPV-positive cervical cancer, both categories are relevant because STAT3 activation is sustained by a network of convergent oncogenic and inflammatory inputs rather than a single pathway abnormality (Strobel et al., 2023).

Most currently available STAT3 inhibitors were originally developed to suppress canonical nuclear signaling (Berkley et al., 2025). For instance, small-molecule inhibitors, such as TTI-101, directly bind the SH2 domain, preventing Tyr705 phosphorylation and halting nuclear entry (Berkley et al., 2025; Li and Dong, 2024). This is important because nuclear STAT3 promotes proliferation, treatment resistance, immune evasion, and anti-apoptotic transcriptional programs (Berkley et al., 2025). However, within the present framework, STAT3-directed therapy may also influence mitochondrial redox and bioenergetic control (Lahiri et al., 2021). Reducing overall STAT3 activity may weaken the signaling environment that favors Ser727-associated outputs and mitochondria-associated STAT3 functions, thereby destabilizing the broader adaptive network that supports redox tolerance under therapy-induced stress (Lahiri et al., 2021).

In this context, strategies that affect Ser727-associated signaling may be particularly relevant (Lahiri et al., 2021). Agents such as napabucasin and Stattic have been reported to influence both canonical and stress-associated STAT3 signaling, thereby affecting not only transcriptional activity but also mitochondria-associated stress responses (Froeling et al., 2019; Löcken et al., 2018; Xia et al., 2021; Wan et al., 2025). In parallel, emerging therapeutic modalities are being developed to more directly address the mitochondrial compartment (Wan et al., 2025). Compounds such as the phytochemical derivative sculponeatin A and mitochondria-targeting synthetic agents, including Mitocur analogs, suggest that mitoSTAT3-associated functions may also be pharmacologically tractable (Wan et al., 2025; Kanchi et al., 2019).

From a therapeutic standpoint, this suggests that STAT3 inhibition should not be viewed only as an anti-proliferative approach. In HPV-positive cervical cancer, STAT3-targeted strategies may also function as modulators of mitochondrial redox fitness, especially when used in combination with therapies that impose oxidative stress (Liu Y. et al., 2023; Strobel et al., 2023; Lahiri et al., 2021). DHODH targeting becomes especially relevant in this context, because it provides a complementary point of intervention at the level of compartment-specific mitochondrial protection (Liu Y. et al., 2023).

### 6.3 DHODH inhibition as a strategy to overcome mitochondrial ferroptosis resistance

DHODH is especially relevant because it links metabolism to mitochondrial ferroptosis defense (Liu Y. et al., 2023; Lee and Roh, 2025; Mishima et al., 2023). As an IMM enzyme, DHODH transfers electrons from dihydroorotate to the CoQ pool, thereby contributing to local CoQH_2_ generation (Lee and Roh, 2025; Mishima et al., 2023; Zeng et al., 2024). In practical terms, this links pyrimidine metabolism to radical-trapping activity within the IMM, where LPO may become especially consequential under chronic oxidative stress (Lee and Roh, 2025; Ma et al., 2022; Tang et al., 2021). When DHODH activity is reduced, tumor cells may lose part of their mitochondrial capacity to restrain lipid radical propagation (Mishima et al., 2023; Ma et al., 2022; Zeng et al., 2024). Consequently, the massive oxidative stress generated by standard CCRT may overwhelm this compromised local mitochondrial protection, driving mitochondrial LPO and terminal ferroptotic injury (Jiang et al., 2023; Mao et al., 2026; Amos et al., 2023). In this sense, DHODH inhibition can be viewed not only as a metabolic intervention but also as a ferroptosis-sensitizing strategy (Lee and Roh, 2025; Ma et al., 2022; Jiang et al., 2023). To exploit this vulnerability, several classes of pharmacological agents are currently being evaluated, ranging from repurposed autoimmune drugs to potent experimental anticancer compounds, such as brequinar (Lee and Roh, 2025; Zhou et al., 2021).

HPV-positive cervical cancer may be especially sensitive to this strategy (Chang and Miao, 2023; Strobel et al., 2023; Jiang et al., 2023). Rather than acting as a universally toxic trigger, DHODH inhibition may sensitize tumors that already operate near the limits of mitochondrial redox tolerance (Liu Y. et al., 2023; Lee and Roh, 2025). Its utility may be even greater when considered within a broader mitochondrial defense network that includes ETC-dependent CoQ cycling and mitoSTAT3-associated bioenergetic regulation (Liu Y. et al., 2023; Lahiri et al., 2021). DHODH inhibition is unlikely to achieve maximal efficacy alone in all tumors (Liu Y. et al., 2023; Mishima et al., 2023). Its effects may be shaped by parallel antioxidant systems and by variations in baseline mitochondrial dependency (Mishima et al., 2023; Wang and Min, 2021; Zeng et al., 2024). This highlights the importance of rational combinations (Lee and Roh, 2025; Su et al., 2022). For instance, preclinical models suggest that the efficacy of competitive inhibitors, such as brequinar, is heavily influenced by baseline GPX4 expression (Wang and Min, 2021; Tang et al., 2021; Olsen et al., 2022); in GPX4-high tumors, the induction of terminal ferroptosis often requires the synergistic co-administration of DHODH inhibitors with agents that impose massive exogenous oxidative stress (Mishima et al., 2023; Ma et al., 2022; Tang et al., 2021; Olsen et al., 2022). When combined with therapies that elevate oxidative stress, DHODH inhibition may amplify the accumulation of mitochondrial LPO and thereby enhance treatment response (Jiang et al., 2023; Mao et al., 2026; Amos et al., 2023). Such combinations may include CCRT, ferroptosis-inducing agents, or interventions that weaken parallel redox-adaptive pathways, including STAT3-associated signaling (Liu Y. et al., 2023; Yong et al., 2024; Jiang et al., 2023).

Pharmacologic interpretation also requires caution. Some DHODH-targeting compounds may have off-target effects, and apparent ferroptosis sensitization may not always reflect DHODH blockade alone (Mishima et al., 2023). To address these pharmacologic limitations, next-generation inhibitors with improved target selectivity and pharmacokinetic profiles are advancing through clinical development (Lee and Roh, 2025; Zhou et al., 2021). Drug repurposing may also broaden the therapeutic options for DHODH targeting. For example, the anthelmintic flubendazole has been reported to promote DHODH protein degradation rather than simply inhibit its enzymatic activity (Lee and Roh, 2025). For this reason, translational development should be supported by orthogonal validation, including genetic perturbation, dose-controlled pharmacology, and compartment-resolved readouts of LPO (Liu Y. et al., 2023; Lee and Roh, 2025). Even with these caveats, DHODH remains one of the most concrete therapeutic entry points within the mitoSTAT3-centered framework (Liu Y. et al., 2023; Lee and Roh, 2025; Jiang et al., 2023). Representative therapeutic strategies targeting the mitoSTAT3–DHODH ferroptosis-defense network are summarized in Table 4.

**TABLE 4:** Therapeutic strategies targeting the mitoSTAT3-DHODH ferroptosis-defense network in HPV-positive cervical cancer.

Target class	Representative agents	Direct or indirect relevance	Evidence stage	Known limitations
Upstream STAT3 pathway inhibitors	Ruxolitinib; gefitinib; erlotinib (Valle‐Mendiola et al., 2023; Gutiérrez‐Hoya and Soto‐Cruz, 2020; Chen et al., 2023b)	Indirect reduction of STAT3-activating inputs	Approved/clinical agents in other settings; cervical cancer use remains contextual	Limited specificity for mitoSTAT3; pathway redundancy and toxicity may occur
Direct canonical STAT3 inhibitors	TTI-101; OPB-51602; OPB-31121 (Berkley et al., 2025; Li and Dong, 2024; Beebe et al., 2018; Brambilla et al., 2015; Tsimberidou et al., 2025)	Directly suppress total/canonical STAT3 signaling; may indirectly reduce mitoSTAT3 fraction	Investigational or early clinical/preclinical	Primarily designed for nuclear STAT3; mitochondrial effects require validation
Broader or dual STAT3 modulators	Napabucasin; Stattic; SLSI-1216 (Froeling et al., 2019; Wan et al., 2025; Genini et al., 2017)	May affect canonical and stress-associated STAT3 outputs including Ser727-linked signaling	Mostly preclinical/early translational	Off-target effects and incomplete selectivity remain concerns
Mitochondria-relevant STAT3-directed agents	Sculponeatin A; Mitocur analogs (Wan et al., 2025; Kanchi et al., 2019; Genini et al., 2017; Pavlyuchenkova et al., 2023)	More direct interference with mitoSTAT3-associated bioenergetic adaptation	Preclinical	Limited disease-specific data; pharmacokinetics, specificity, and toxicity require evaluation
Repurposed DHODH inhibitors	Leflunomide; teriflunomide (Zhou et al., 2021; Sainas et al., 2018)	Direct DHODH inhibition and reduction of DHODH-linked CoQH_2_ generation	Clinically used in non-oncology indications; anticancer use investigational	Immunomodulatory effects, systemic toxicity, and dosing constraints
Selective or next-generation DHODH inhibitors	Brequinar; AG-636; vidofludimus calcium; JBZ-001 (Lee and Roh, 2025; Mishima et al., 2023; Zhou et al., 2021; Sainas et al., 2018)	Direct suppression of DHODH-dependent mitochondrial redox protection and pyrimidine synthesis	Preclinical to clinical depending on agent	Potential off-target effects; response depends on parallel ferroptosis defenses
Alternative DHODH-disrupting approaches	Flubendazole (Lee and Roh, 2025)	Promotes DHODH loss or disruption beyond conventional enzymatic inhibition	Preclinical/repurposing	Mechanism and selectivity require orthogonal validation
Combination strategies	STAT3 inhibitor + DHODH inhibitor; CCRT + DHODH inhibitor; STAT3 inhibitor + CCRT (Chang and Miao, 2023; Liu et al., 2023a; Yong et al., 2024; Jiang et al., 2023; Amos et al., 2023; Su et al., 2022; Olsen et al., 2022)	Simultaneous weakening of adaptive signaling and local ferroptosis defense	Preclinical rationale	Requires biomarker selection, dosing optimization, and toxicity monitoring

### 6.4 Nanomedicine-enabled delivery and co-delivery strategies

Nanomedicine offers a practical strategy to translate this mechanistic framework into clinical practice. STAT3-targeting agents and redox-modulating compounds face similar pharmacological limitations such as limited solubility, low bioavailability, and off-target toxicity (Pindiprolu et al., 2018; Ashrafizadeh et al., 2019). Nanoformulations can help address these issues by improving stability, prolonging circulation, increasing tumor accumulation, and enabling stimulus-responsive release (Ashrafizadeh et al., 2019). For example, pH-sensitive N-methacrylamide copolymer conjugates have been engineered to release STAT3 inhibitors within the acidic tumor microenvironment (Kovář et al., 2023). This design may reduce systemic exposure and toxicity (Kovář et al., 2023).

Another advantage of nanomedicine is co-delivery of multiple therapeutic agents, enabling synergistic effects in cancer therapy (Sun et al., 2024; Li et al., 2024; Bostami et al., 2022). Combined targeting of STAT3 signaling and DHODH-dependent mitochondrial protection may be more effective than either approach alone. Nanoparticle-based platforms can co-deliver a STAT3 inhibitor with a DHODH-targeting agent or integrate one of these with a ROS-amplifying payload (Kovář et al., 2023; Kostka et al., 2022). For instance, preclinical studies have shown that polymeric nanoparticles co-delivering doxorubicin and STAT3 inhibitors can achieve synchronized release and help overcome multidrug resistance (Kovář et al., 2023; Kostka et al., 2022; Misra et al., 2019). Cationic solid lipid nanoparticles can protect fragile STAT3 siRNA or shRNA to achieve genetic silencing alongside conventional chemotherapy (Pindiprolu et al., 2018; Kang et al., 2024). Such designs are particularly relevant to HPV-positive cervical cancer. In this setting, tumor persistence may depend on both sustained STAT3 signaling and mitochondrial redox adaptation under chronic oxidative stress (Chang and Miao, 2023; Tiwary et al., 2025). Mitochondria-directed or redox-responsive delivery systems may further improve specificity by concentrating active agents near vulnerable mitochondrial membranes (Liu Y. et al., 2023). By utilizing surface functionalization with lipophilic cations, such as triphenylphosphonium, nanocarriers can exploit the highly negative mitochondrial membrane potential to deposit DHODH inhibitors or mitoSTAT3 degraders directly at the IMM (Pawar et al., 2022; Liu et al., 2024; Dou et al., 2022).

The true translational potential of the mitoSTAT3-DHODH network is to combine these targeted inhibitors with established clinical regimens. Because CCRT already imposes substantial oxidative and membrane stress, it may pair well with strategies that weaken mitochondrial defenses (Chang and Miao, 2023; Jiang et al., 2023). Chemoradiotherapy provides oxidative pressure, whereas STAT3-and DHODH-directed interventions may lower the barrier to mitochondrial failure (Liu Y. et al., 2023). However, this route is more complex, since ferroptosis-sensitizing approaches and excessive oxidative injury may impair CD8-positive T-cell function if applied indiscriminately (Drijvers et al., 2020; Jang et al., 2025; Diao et al., 2024).

### 6.5 Combination therapy, biomarkers, and safety considerations

The translational value of the mitoSTAT3–DHODH framework is rational combination therapy rather than monotherapy (Li et al., 2025b; Jiang et al., 2023). In HPV-positive cervical cancer, CCRT imposes substantial oxidative and membrane stress (Chang and Miao, 2023). STAT3-and DHODH-directed interventions may enhance treatment response not simply by adding cytotoxicity, but by weakening the mitochondrial defense systems that allow tumor cells to tolerate treatment-induced oxidative damage (Jiang et al., 2023). In this sense, CCRT provides oxidative pressure, whereas disruption of mitoSTAT3-associated adaptation or DHODH-dependent mitochondrial protection may lower the barrier to mitochondrial failure (Liu Y. et al., 2023; Jiang et al., 2023).

Combining this approach with immunotherapy may also be important (Li et al., 2025b). STAT3 contributes to immune evasion, cytokine remodeling, and maintenance of an immunosuppressive tumor microenvironment (Rossetti et al., 2020; Noh et al., 2022; Shi et al., 2021). STAT3 inhibition may help improve antitumor immune activity while weakening tumor-intrinsic survival programs (Strobel et al., 2023; Rossetti et al., 2020; Noh et al., 2022). Ferroptosis-sensitizing approaches should be integrated carefully because excessive oxidative injury may compromise the viability and function of CD8^+^ T cells and other antitumor immune populations (Drijvers et al., 2020; Jang et al., 2025; Diao et al., 2024). The goal should be to exploit tumor redox vulnerability while preserving immune function through selective delivery and rational scheduling (Li et al., 2025b). Ferroptosis-sensitizing approaches should be integrated carefully because excessive oxidative injury may compromise the viability and function of CD8^+^ T cells and other antitumor immune populations (Drijvers et al., 2020; Jang et al., 2025; Diao et al., 2024). The goal should be to exploit tumor redox vulnerability while preserving immune function through selective delivery and rational scheduling (Li et al., 2025b).

These safety and efficacy concerns make biomarker-guided patient selection important (Li et al., 2025b). However, not all HPV-positive cervical cancers equally rely on STAT3 signaling, mitochondrial redox adaptation, or ferroptosis defense (Chang and Miao, 2023). Relevant biomarker classes may include phosphorylated STAT3 (pSTAT3), particularly Ser727-associated signaling (Shi et al., 2021); redox- and ferroptosis-related markers, such as GPX4, SLC7A11, and DHODH (Chang and Miao, 2023; Li et al., 2025b; Ning et al., 2023); and pharmacodynamic readouts of LPO, including 4-hydroxynonenal, malondialdehyde, or compartment-resolved mitochondrial oxidative-stress markers (Li et al., 2025b). In practice, patient stratification will likely require an integrated assessment of pathway activation, ferroptosis-defense status, and treatment context rather than reliance on a single marker. Candidate biomarkers and pharmacodynamic readouts for ferroptosis-directed patient stratification and treatment monitoring are summarized in Table 5.

**TABLE 5:** Biomarkers and pharmacodynamic readouts relevant to ferroptosis-directed patient stratification and treatment monitoring.

Biomarker category	Representative markers or assays	Clinical validation status	Evidence level	Translational relevance
STAT3 pathway activation	Total STAT3; pSTAT3; pS727-STAT3; pY705-STAT3; IL-6/JAK/STAT signatures (Strobel et al., 2023; Valle‐Mendiola et al., 2023; Gutiérrez‐Hoya and Soto‐Cruz, 2020; Shi et al., 2021)	Exploratory for this framework	Clinical association/preclinical mechanism	May identify tumors with sustained STAT3 signaling and stress-associated outputs
Ferroptosis-defense capacity	GPX4; SLC7A11; FSP1 [2,16,97–98,152–153]	Not validated for patient selection in this setting	Mechanistically supported	May indicate contribution of parallel antioxidant systems
DHODH-associated mitochondrial protection	DHODH expression; protein-level or transcriptional readouts (Liu et al., 2023a; Lee and Roh, 2025; Mishima et al., 2023; Wang and Min, 2021; Zhou et al., 2021; Jiang et al., 2023)	Exploratory	Preclinical/mechanistic	May identify tumors with functionally relevant mitochondrial CoQ-linked protection
LPO readouts	4-HNE; MDA; LPO staining or biochemical assays (Ayala et al., 2014; Ide and Souma, 2022)	Pharmacodynamic, not specific alone	Preclinical/translational	May provide evidence of oxidative membrane injury during treatment
Mitochondrial oxidative-stress markers	Mitochondrial ROS probes; Mito-BODIPY; compartment-resolved oxidative readouts (Greenwood and Witney, 2021; Iannetti et al., 2019)	Mainly preclinical	Experimental	May monitor treatment-induced mitochondrial injury
Composite ferroptosis signatures	Ferroptosis gene panels; integrated transcriptional risk models (Han et al., 2023)	Exploratory	Bioinformatic/clinical association	May improve stratification beyond single-marker assessment
Exploratory structural readouts	Transmission electron microscopy; mitochondrial morphology (Liu et al., 2023a; Ide and Souma, 2022)	Preclinical/supportive	Experimental	May support evidence of mitochondrial damage in early studies

## 7 Future validation strategies and translational challenges

As the mitoSTAT3-ETC-DHODH connection remains a proposed working model, rigorous experimental validation is essential (Liu Y. et al., 2023; Lahiri et al., 2021). Priority should be given to paired HPV-positive and HPV-negative cervical cancer models (Chang and Miao, 2023; Li et al., 2025b), patient-derived organoids, and xenograft systems that permit direct comparison of viral status, mitochondrial dependency, and ferroptosis sensitivity (Lõhmussaar et al., 2021; Liu L. et al., 2023).

Mechanistic validation should combine genetic perturbation and metabolic readouts (Liu Y. et al., 2023; Lahiri et al., 2021; Lee and Roh, 2025; Pallotti et al., 2021; Jiang et al., 2023). Relevant approaches include STAT3 knockdown or knockout, Ser727 phospho-mutant rescue experiments, mitoSTAT3 localization assays, DHODH knockdown or knockout, dose-controlled DHODH inhibition, and rescue experiments using ferroptosis suppressors or CoQ-linked interventions (Lee and Roh, 2025; Mohammed et al., 2020; Mishima et al., 2023). These studies should be paired with Seahorse-based respiratory profiling, CoQ/CoQH_2_ redox measurements, isotope tracing of DHODH-linked flux, and compartment-resolved ROS or LPO assays such as Mito-BODIPY, mitochondrial 4-HNE imaging, or mitochondrial ROS probes (Greenwood and Witney, 2021; Iannetti et al., 2019).

Clinical translation will require careful biomarker validation (Li et al., 2025b; Shi et al., 2021; Ning et al., 2023). Candidate markers such as pS727-STAT3, total and phosphorylated STAT3, DHODH, GPX4, SLC7A11, FSP1, 4-HNE, MDA, and mitochondrial oxidative-stress readouts should be tested in clinically annotated cervical cancer patient cohorts (Li et al., 2025b; Greenwood and Witney, 2021; Iannetti et al., 2019). As validated cutoff values have not yet been established, future studies should link marker levels to treatment response, recurrence, survival, and pharmacodynamic evidence of LPO induction (Li et al., 2025b; Shi et al., 2021; Ning et al., 2023). Selective delivery, rational scheduling, and immune-preserving combination design will also be necessary to reduce off-target toxicity and avoid compromising antitumor immune cells (Drijvers et al., 2020; Jang et al., 2025; Diao et al., 2024).

## 8 Conclusions and perspectives

HPV-positive cervical cancer develops under persistent metabolic, replicative, and oxidative stress (Chang and Miao, 2023), and this burden is further intensified by cisplatin-based chemoradiotherapy (Jiang et al., 2023). In this context, therapeutic resistance is unlikely to be explained solely by genomic alterations or generalized antioxidant capacity. Rather, current evidence supports a model in which tumor survival depends, in part, on compartment-specific defense systems that preserve mitochondrial integrity and restrain LPO under chronic stress (Li et al., 2025b; Liu Y. et al., 2023).

STAT3 should be viewed as both a canonical nuclear transcription factor and a stress-adaptive signaling mediator with mitochondria-associated functions (Liu Y. et al., 2023). Emerging evidence suggests that mitoSTAT3 may support ETC efficiency and limit electron leakage (Liu Y. et al., 2023; Lahiri et al., 2021; Comità et al., 2021; Ploeger et al., 2020). DHODH links pyrimidine metabolism to local CoQH_2_ generation within the IMM and may provide a compartment-specific mitochondrial defense layer against ferroptotic injury (Li et al., 2025b; Liu Y. et al., 2023; Jiang et al., 2023). Together, these observations support the mitoSTAT3–DHODH axis as a mechanistically plausible framework for understanding how HPV-positive cervical cancer cells tolerate sustained oxidative pressure (Liu Y. et al., 2023; Wang and Min, 2021).

Rather than simply increasing oxidative damage, more effective strategies may focus on weakening mitochondrial adaptive systems that allow tumor cells to survive treatment-induced stress (Liu Y. et al., 2023). STAT3-targeted approaches and DHODH inhibition should be considered not only as independent antitumor strategies but also as potential sensitizers that reduce the ability of tumor cells to withstand chemoradiotherapy-associated membrane injury (Jiang et al., 2023). Their value may be apparent in rational combinations, particularly when integrated with standard CCRT (Li et al., 2025b; Jiang et al., 2023). Successful translation of this framework will likely depend on biomarker-guided patient selection, rational combination design, and nanomedicine-enabled delivery strategies that improve tumor selectivity while limiting systemic toxicity (Li et al., 2025b).

In summary, the mitoSTAT3-DHODH axis provides a mechanistically plausible framework for understanding ferroptosis resistance and therapy tolerance in HPV-positive cervical cancer. Future studies should prioritize paired HPV-positive and HPV-negative models, STAT3 and DHODH genetic perturbation, Ser727 mutant rescue experiments, direct CoQ/CoQH_2_ redox measurements, compartment-specific LPO assays, patient-derived organoids, xenograft models, and biomarker validation in clinically annotated cohorts. A deeper understanding of this network may create new opportunities for biomarker-guided, combination-based, and nanomedicine-enabled treatment strategies that exploit mitochondrial vulnerability while preserving normal tissue and immune function.
